# SEA version 3.0: a comprehensive extension and update of the Super-Enhancer archive

**DOI:** 10.1093/nar/gkz1028

**Published:** 2019-10-31

**Authors:** Chuangeng Chen, Dianshuang Zhou, Yue Gu, Cong Wang, Mengyan Zhang, Xiangyu Lin, Jie Xing, Hongli Wang, Yan Zhang

**Affiliations:** 1 School of Life Science and Technology, Computational Biology Research Center, Harbin Institute of Technology, Harbin 150001, China; 2 College of Bioinformatics Science and Technology, Harbin Medical University, Harbin 150081, China

## Abstract

Super-enhancers (SEs) are critical for the transcriptional regulation of gene expression. We developed the super-enhancer archive version 3.0 (SEA v. 3.0, http://sea.edbc.org) to extend SE research. SEA v. 3.0 provides the most comprehensive archive to date, consisting of 164 545 super-enhancers. Of these, 80 549 are newly identified from 266 cell types/tissues/diseases using an optimized computational strategy, and 52 have been experimentally confirmed with manually curated references. We now support super-enhancers in 11 species including 7 new species (zebrafish, chicken, chimp, rhesus, sheep, Xenopus tropicalis and stickleback). To facilitate super-enhancer functional analysis, we added several new regulatory datasets including 3 361 785 typical enhancers, chromatin interactions, SNPs, transcription factor binding sites and SpCas9 target sites. We also updated or developed new criteria query, genome visualization and analysis tools for the archive. This includes a tool based on Shannon Entropy to evaluate SE cell type specificity, a new genome browser that enables the visualization of SE spatial interactions based on Hi-C data, and an enhanced enrichment analysis interface that provides online enrichment analyses of SE related genes. SEA v. 3.0 provides a comprehensive database of all available SE information across multiple species, and will facilitate super-enhancer research, especially as related to development and disease.

## INTRODUCTION

Super-enhancers (SEs) are enhancer clusters bound by master transcription factors. SEs affect the transcriptional activation of most genes, and participate in disease development, cell differentiation and tissue type identity specification. SEs differ from typical enhancers in terms of a larger size and transcription factor density, a tendency to strongly activate transcription and robustness to perturbation (1). SEs can regulate gene expression and specify cell type (2–4). SEs play key roles in cancer cell maintenance and impact oncogene transcriptional processes. Cancer cell dependence on SE transcriptional and proliferative activities may provide a fatal weakness in targeted-cell therapy design. Understanding the content and mechanism of the SE complex will facilitate the study of drug-targeted-cell cancer therapy (5).

Several methods are available for SE identification, including computational and experimental technologies (6). Computational methods can identify a large number of SEs in a short time, whereas experimental methods can clarify SE mechanism and activity (7). Studies have shown that nucleosomes with the histone modification H3K27ac are enriched at active enhancers (8,9). H3K27ac datasets identified using Chromatin Immunoprecipitation sequencing (ChIP-Seq) helped identify the majority of known SEs; the modification seems to be a signature of SEs (3), as well as Med1 (10). Moreover, BRD4, which belongs to the bromodomain and extra-terminal domain (BET) family, has been identified in many studies as an epigenetic regulator that can affect cell transcription (11). The coactivator p300 is a histone acetyl-transferase that can recruit the transcription initiation complex to initiate transcription and may impact protein ubiquitination modification and degradation (12,13). H3K27ac modification, BRD4, Med1 and p300 associations, are all enriched in SEs compared with typical enhancers, as shown by ChIP-Seq data. We consider the existence of these four factors to be an essential signature for computationally recognizing SEs. The rapid growth of publicly available H3K27ac, BRD4, Med1 and p300 ChIP-Seq datasets stored in Gene Expression Omnibus (GEO), Sequence Read Archive (SRA) and ENCODE (14) provides an excellent opportunity to computationally identify SEs in multiple cell types and tissues using ChIP-Seq datasets.

Researchers have developed several databases for the storage of SE data in the past. In 2015, we proposed and built a database named the Super Enhancer Archive (SEA v. 1.0 http://sea.edbc.org), with the aim to provide a comprehensive archive of super-enhancers in numerous species (15). dbSUPER provides a SE list from the mouse and human genome (16). SEdb is designed to store resources on human SEs (17), and SELER recognizes SE associated lncRNA in human cancer cell lines (18). Most of these functions were designed to computationally identify and store SEs, but not for the functional analysis of SEs. To this end, we updated the Super Enhancer Archive to version 3.0 (SEA v. 3.0, http://sea.edbc.org), which integrates SE data with regulatory elements, and provides comprehensive annotation regarding the formation and potential roles in the regulation of cell identity and associated effects after targeted editing. SEA v. 3.0 stores the SEs and conventional enhancers of 266 cell types, tissues and diseases across 11 species; provides optimized query criteria to comprehensive SE information; provides an updated multi-omics visualization genome browser; evaluates the specificity of SE cell type by Shannon Entropy; and provides the Enrichr enrichment analysis interface (19) for SE related genes. Additionally SEA v. 3.0 provides comprehensive regulatory element access. This includes ref-genes; CpG islands; H3K27ac, BRD4 and p300 landscapes; SE constituents; methylation and expression levels; transcription factor binding sites; relative conservation across the 11 species; SpCas9 target sites; SNP sites; and spatial interactions by High-throughput/resolution chromosome conformation capture (Hi-C); all available for customized data visualization across multiple cell types, tissues and disease states. In brief, SEA v. 3.0 provides a comprehensive platform for the storage, annotation, query, functional analysis and visualization of SEs.

## DATA EXPANSION AND PRE-PROCESSING

**Figure 1.**
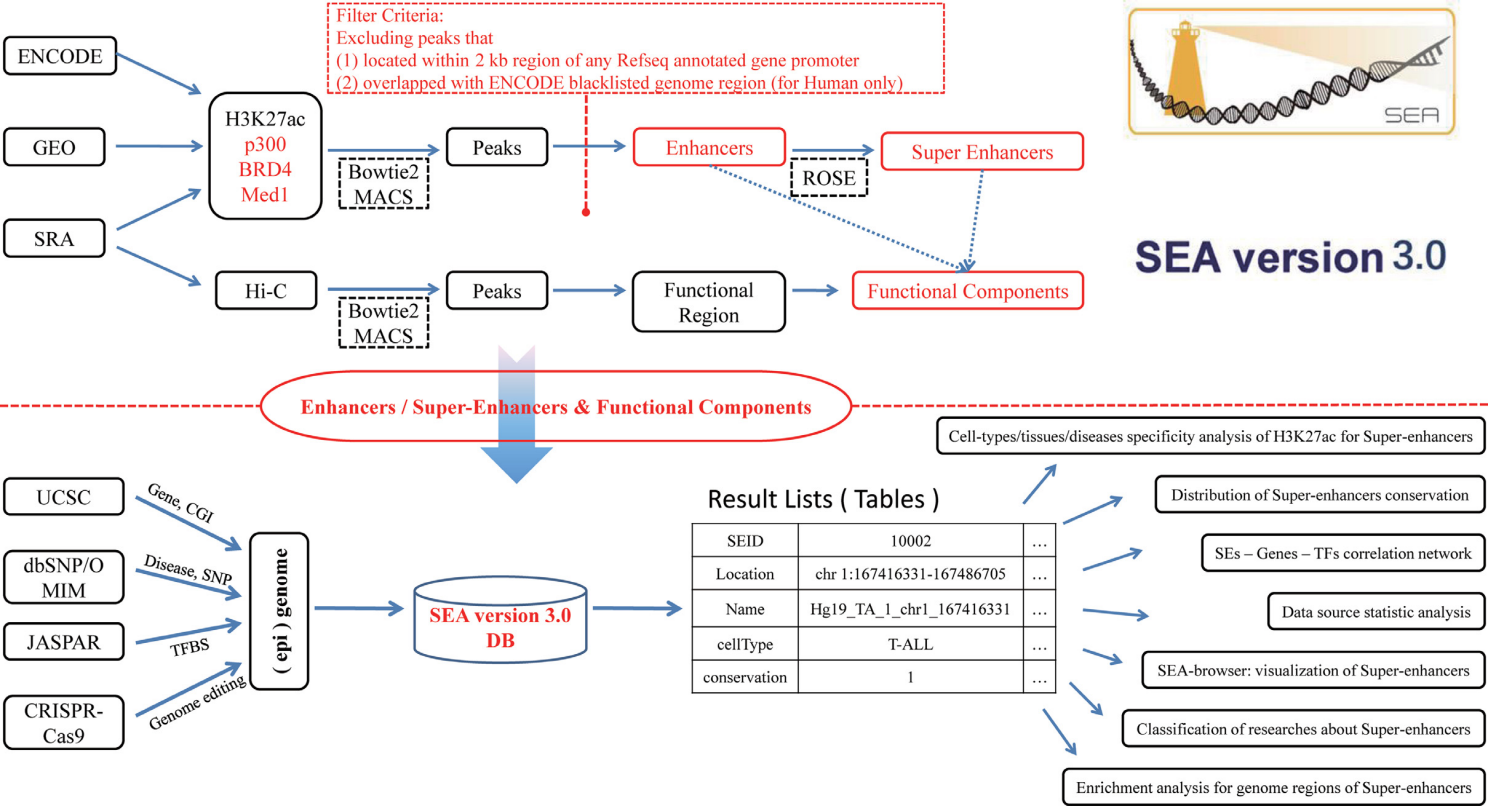
Database content and construction. SEA v. 3.0 takes advantage of available public H3K27ac, BRD4, Med1 and p300 ChIP-Seq datasets to identify super-enhancers in different cell types/tissues/diseases of 11 species. It excludes peaks located within ±2 kb of any RefSeq annotated gene promoter or peaks overlapping with ENCODE blacklisted genomic regions. Multiple track types are used for genomic visualization including functional components generated by Hi-C datasets. Shannon Entropy is used to calculate and evaluate the cell type specificity of super-enhancers, and all data are accessible through the download page.

SEA v3.0 is a comprehensive database that provides criteria query, genome browser, personalized analysis tools and data downloads of SEs. An overview of SEA v3.0 is shown in Figure 1. H3K27ac, BRD4, Med1 and p300 datasets by ChIP-Seq of 266 cell types/tissues/diseases for 11 species were collected from the public ENCODE, GEO and SRA databases. This is a major expansion of the previous version, which contained 134 cell types in four species, with only H3K27ac ChIP-Seq datasets used for SE identification. Bowtie2 (v. 2.2.5) (20) and ROSE (21) were used to map ChIP-Seq reads to reference genomes and obtain candidate SEs for SEA v. 3.0 for 11 species: human (hg38), mouse (mm10), *D. melanogaster* (dm6), *C. elegans* (ce10), zebrafish (danRer11), chicken (galGal5), chimp (panTro5), rhesus (rheMac8), sheep (oviAri3), xenopus tropicalis (xenTro9) and stickleback (gasAcu1). Peaks located within a ±2 kb region of any RefSeq annotated gene promoter, or that overlapped with any ENCODE blacklisted genomic regions were excluded.

In this way, we incorporated 164 545 SEs and 3 361 785 enhancers computationally predicted in 266 cell types, tissues or diseases from 11 species. In detail, for human, 109 447 SEs were computationally predicted in 133 cell types, including 93 870 identified by H3K27ac, 10437 by p300, 4195 by BRD4 and 942 by Med1. For mouse, 23 964 SEs were identified in 39 cell types or tissues, including 19 721 identified by H3K27ac and 4243 by p300. Detailed SE statistics are shown in Supplementary Table S1. In addition, we manually curated experimentally supported SE data by strict experimental method through a review of more than 500 published papers. These papers were collected using keyword ‘super enhancer’ from PubMed. A total of 52 SE-related genes were obtained whose transcription affected by perturbation of relevant SEs (Supplementary Table S2). We provide 610 datasets including the super-enhancer information processed by SEA v. 3.0 and linkage of raw data source that were all reprocessed for super-enhancer identification and genome browser visualization in the download page. Data expansion and updating points are also shown in Table 1.

**Table 1.** SEA v3.0 data content compared with previous version of SEA

Content		SEA	SEA v3.0	Fold increase
Super-enhancers	Species	4	11	2.75
	Recognition factor	H3K27ac	H3K27ac,BRD4,p300,Med1	4
	Super-enhancer	83 996	164 545	1.96
	Enhancer	No	3 361 785	New
	Experimental confirmed SEs	3	52	17.33
	Cell types/tissues/disease	134	266	2.0
Genome browser	DNA methylation	26	37	1.42
	H3K27ac	194	208	1.07
	Expression	35	87	2.49
	TF ChIP-seq data	98	126	1.29
	4D genome	No	Yes	New
	SE constituent	No	32	New
	Reference genome	4	11	2.75
	CpG islands	4	11	2.75
	SNP	Yes	Yes	New
	Transcription factor binding sites	Yes	Yes	New
	CRISPR-Cas9	Yes	Yes	New
	p300	No	34	New
	BRD4	No	2	New
	Conservation	Yes	Yes	New
	Genome position	Yes	Yes	New
Analysis tools	GREAT	Yes	Yes	-
	Enrichr	No	Yes	New
	Specific analysis of H3K27ac status	Yes	Yes	-
	SE cell type specificity	No	Yes	New
	TF enrichment analysis	Yes	Yes	New
	Regulatory network	Yes	Yes	New
	Query	Yes	Yes	New
	Data Downloads	Yes	Yes	New
Others	Publications related to super-enhancers	8	52	6.5

## UPDATE OF DATABASE MODULES

### Searching engine update

**Figure 2.**
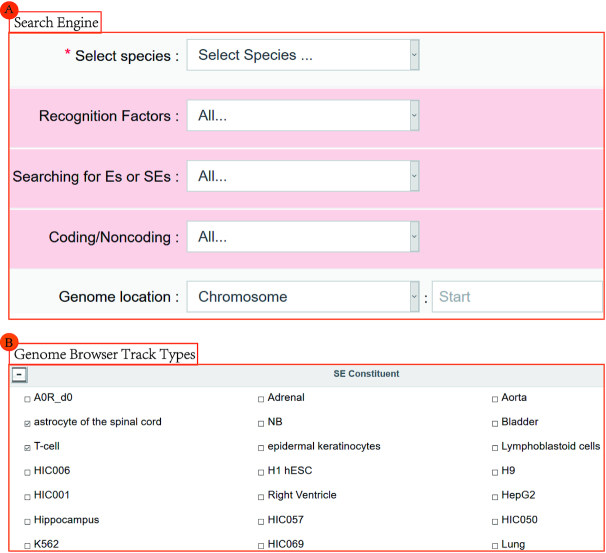
SEA v. 3.0 update modules. (**A**) Searching engine updates added three query options. (**B**) New track types updates include SE constituent computed by Hi-C in multiple cell types and 4D Genome.

Our search engine was enhanced in SEA v. 3.0 to support additional accuracy. In addition to our original query options (species, genome location, gene name, cell types or tissues, SE name and transcription factors), three new options namely ‘recognition factors’, ‘searching for Es or SEs’ and ‘Coding/Noncoding’ have been added to help users query the specifying SEs with coding or noncoding related genes (Figure 2A). The results are displayed on a search result page that contains, the SEID (the identity number of SE), SE genomic loci, SE name and associated recognition factors. A new tool, Enrichr, was added for enrichment analysis using SE related genes, by which users can generate a download page of SE related genes or redirect those genes to the Enrichr database for enrichment analysis. GREAT (22) analysis of specified SEs is also provided in SEA v. 3.0.

### Genome browser update

Nearby genes and SEs overlapped with user-specified genomic regions are presented in the SEA browser pages, together with related CpG islands, nucleotide conservation among 11 species and chromosomal location, all visualized by default. Furthermore, the H3K27ac modification states of 208 cell types, DNA methylation of 37 cell types, mRNA level of 87 cell types, SNPs, transcription factor binding measured by ChIP-Seq of 126 cell types and CRISPR/Cas9 target sites in the input genomic regions can be visualized manually. The BRD4-binding sites in two cell types, and p300-binding sites in 34 cell types are also available for visualization.

Hi-C was designed to capture genome-wide chromatin interactions and reveal the 3D structure of the genome; this may show possible regulatory interactions between genes. It is based on the cross-linking of DNA fragments with long linear distances but close spatial structure, and then an enrichment of cross-linked DNA fragments, and executed using high-throughput sequencing. We obtained Hi-C datasets of 32 human cell types from GEO. The raw fastq format files were aligned using bowtie2; then peak calling was executed by MACS (23), a software designed for model-based analysis of ChIP-Seq. These datasets are available as the ‘SE Constituent’ browser view track for visualization, and provide hypothetical information regarding genome-wide chromatin interactions within the user specified genomic region. Furthermore, we also integrated the 3C, 4C, 5C, ChIA-PET, Capture-C and IM-PET data from the 4DGenome project. These data can be visualized as the ‘4DGenome’ browser view track to inquire about chromatin spatial interactions (Figure 2B).

### Enhanced functional analysis tools

Three existing online SE analysis tools were further enhanced in SEA v. 3.0 that (i) builds bed files of specified SEs for download and GREAT analysis; (ii) calculates H3K27ac status specificity, which is represented by the mean value of histone modifications in the SE regions across the selected cell types; (iii) performs enrichment analysis of transcription factors in specified SEs. Our enhanced functional analysis tools provide powerful and robust performance in annotating SEs across various dimensions.

### Newly developed functional analysis tool for SE cell type specificity

Cell type specificity is prominently characteristic of SEs, and is very important for cell type identity. To facilitate measurement of this feature, a new tool was developed to quantify the specificity of SEs across selected cell types by Shannon Entropy, which was employed to solve the problem of measuring information in a quantitative fashion. We assign:

$$\begin{equation*}{\rm{P}}\left( {{x_i}} \right) = \frac{{{S_i}}}{{{S_n}}}\end{equation*}$$

where ${S_i}$ represents the histone modification level of an SE in the $i{\rm{th}}$ cell type, and ${S_n}$ is the total histone modification level in the selected *n* cell types. Thus, ${\rm{\ P}}( {{x_i}} )$ represents the probability of histone modification in SE of the *i*th cell type among the selected *n* cell types. Building on this premise, we propose:

$$\begin{equation*}{\rm{H}}\left( {\rm{X}} \right) = - \,\mathop \sum \limits_{i\ = \ 1}^n P\left( {{x_i}} \right)log_2^{P\left( {{x_i}} \right)}\end{equation*}$$

where ${\rm{H}}( {\rm{X}} )$ represents Shannon Entropy. The greater the uncertainty of the variable is, the larger the Shannon Entropy generated. Therefore, the closer ${\rm{H}}( X )$ approaches $log_2^n$, the more general that SE is in the selected cell types; otherwise, the closer ${\rm{H}}( X )$ approaches 0, the higher the specificity of that SE is predicted to be.

## A CASE APPLICATION SHOWING SELECT SEA V. 3.0 FEATURES

**Figure 3.**
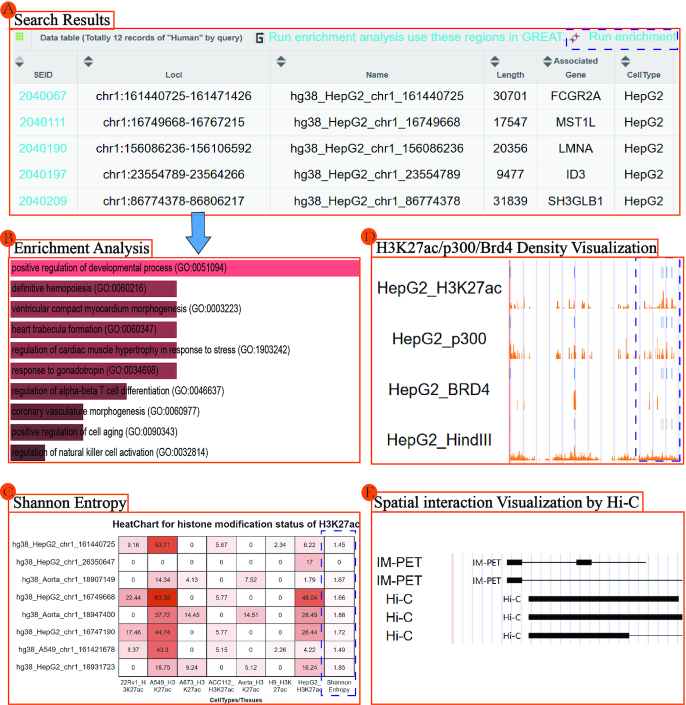
A case application showing select SEA v. 3.0 features. (**A**) Super-enhancers with related coding genes computationally recognized by p300 in chromosome 1 of the human HepG2 cell line. (**B**) Enrichment analysis of super-enhancer related genes through the Enrichr interface. (**C**) Cell type specificity of super-enhancers computed by Shannon Entropy. (**D**) H3K27ac, p300 and Brd4 density of HepG2 super-enhancers visualized in the genome browser. (**E**) Spatial interaction visualization by Hi-C in the genome region ‘chr1:156864585–156975979’.

Chromosome 1 SEs of the human HepG2 cell line that are computationally recognized by p300 were searched, and 12 records were returned (Figure 3A). We then performed enrichment analysis of these SE-related genes using Enrichr through the interface provided on the result page. Enrichr returns multiple types of enrichment information, including transcription, pathways, ontologies and disease/drug interactions. For example, these SE-related genes enrich for the positive regulation of developmental processes, the regulation of α–β T-cell differentiation, and the regulation of natural killer cell activation, which may all potentially affect tumor progression (Figure 3B and Supplementary Figure S1). Next we visualized the genomic region ‘chr1:156086236–256106592,’ which includes two SEs on the search result page. This region shows a high density of H3K27ac, p300, Brd4 and SE constituents in the HepG2 cell line (Figure 3D). We also provide the custom data visualization (Supplementary Figure S2). The spatial interactions of the region by Hi-C or ChIA-PET provide strong evidence for the functional targets of two SEs (Figure 3E). Finally, the cell-type specificity of H3K27ac was calculated by Shannon Entropy and measured for five SEs of HepG2 across 22Rv1, A549, A673, ACC112, Aorta, H9 and HepG2 cell lines. As shown in Figure 3C, A549 has high H3K27ac modification in seven of nine SEs computed, and HepG2 has modification in all nine SEs. Shannon Entropy is calculated for every SE across selected cell types. The more the value close to 0, the more specificity the SE is. Specifically, the HepG2 cell line specific SE shows high cell type specificity (close to 0) and may associate with the development of cancer (Figure 3C and Supplementary Figure S3).

## FUTURE DEVELOPMENT

The importance of SEs is now widely accepted, especially in physiological and pathological processes, such as development and disease. To support the in-depth study of SEs, we will constantly strive to update SEA and improve database functionality to provide a SE database concentrating on multiple species and requirements. Additional information related to SEs will be added to the database as it becomes available. With this continuous data update, SEA is ensured to be timeless. In particular, more detailed cell subtypes are being identified with the rapid development of single-cell research. For example, SE identification based on single-cell ChIP-seq technology is expected in the nearby future. We plan to add more and more -omics data as we update SEA, such that more and more researchers will be able use it.

## Supplementary Material

gkz1028_Supplemental_Files
