# Supplementary material for: SEA version 3.0: a comprehensive extension and update of the Super-Enhancer archive
Source: Nucleic Acids Res. 2019 Oct 31;48(D1):D198–203. doi: 10.1093/nar/gkz1028 (PMC7145603; doi:10.1093/nar/gkz1028)
Supplement: gkz1028_Supplemental_Files [file gkz1028_supplemental_files.zip › Supplementary material.pdf]

Supplementary materials of “SEA version 3.0: a comprehensive extension and update of the Super-Enhancer”

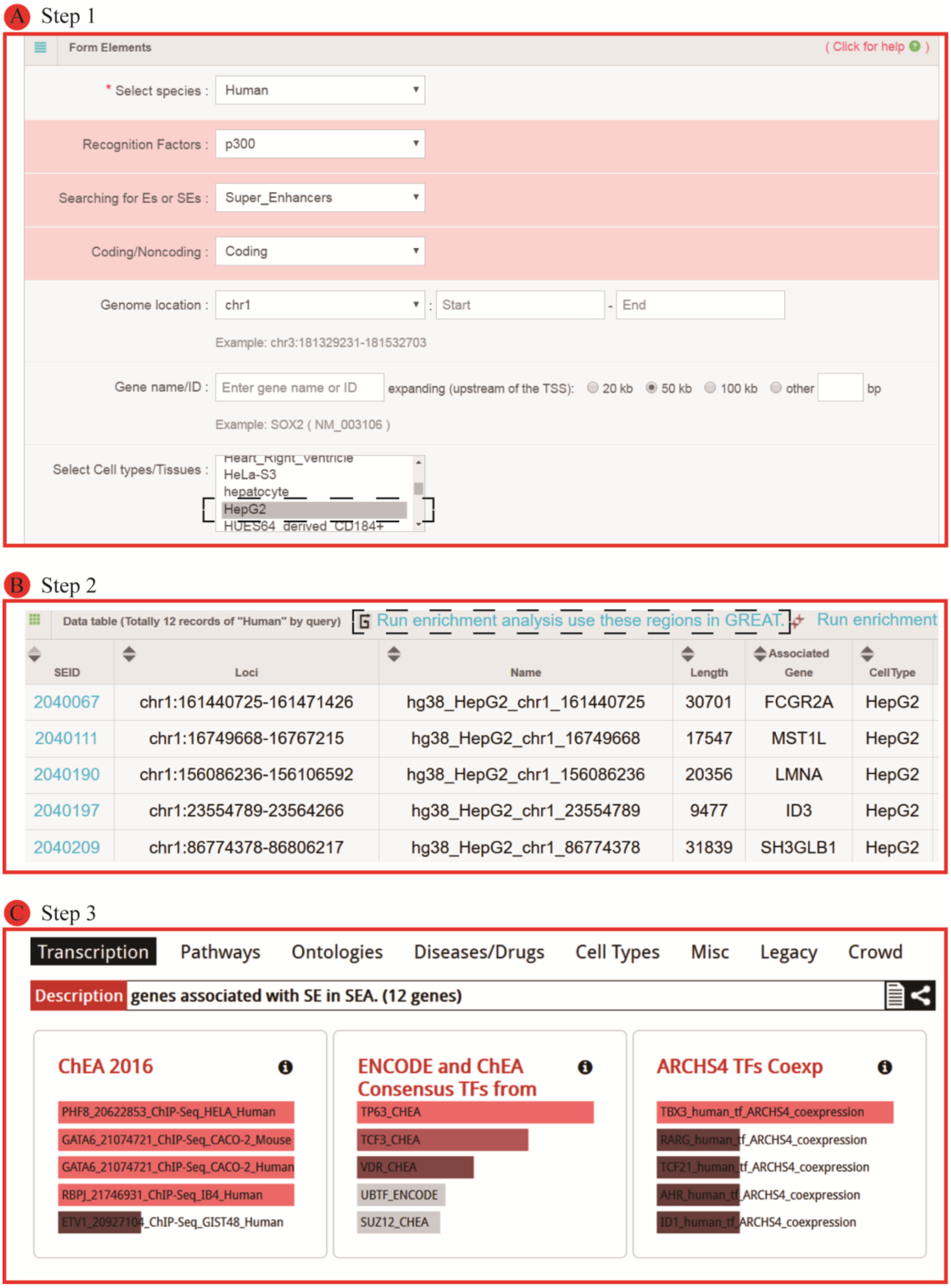

Supplementary Figure S1. Enrichment analysis of super-enhancer related genes. (A). condition query for super-enhancers. (B) Enrichment analysis of Enrichr interface for super-enhancer related genes. (C) Enrichment Analysis result for super-enhancer related genes.

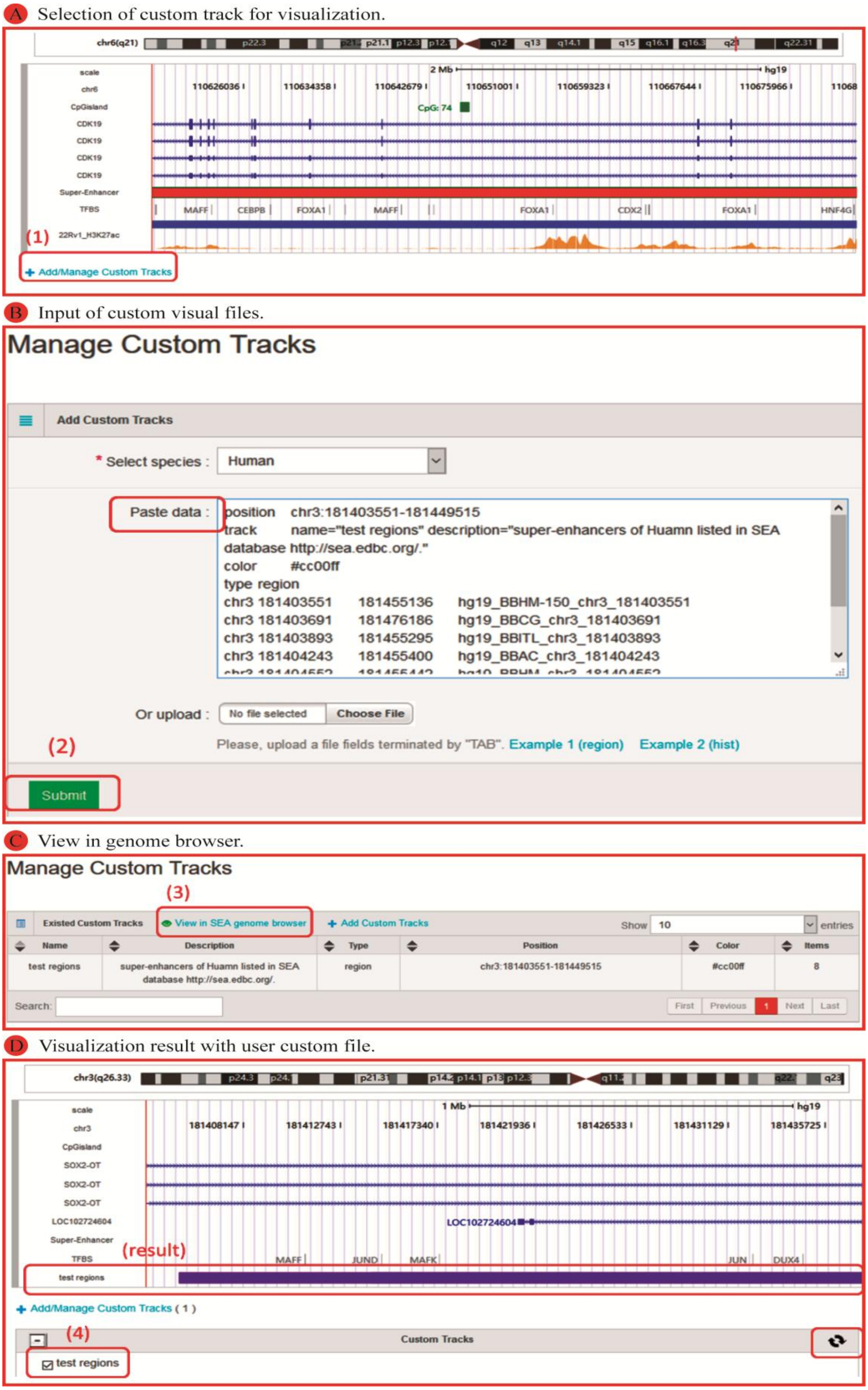
A Selection of custom track for visualization.
chr6(q21)
scale
chr6
CpGisland
CDK19
CDK19
CDK19
CDK19
Super-Enhancer
TFBS
22Rv1_H3K27ac
2 Mb
hg19
CpG: 74
(1)
+ Add/Manage Custom Tracks
B Input of custom visual files.
Manage Custom Tracks
Add Custom Tracks
* Select species :
Human
Paste data :
position chr3:181403551-181449515
track name="test regions" description="super-enhancers of Huamn listed in SEA database http://sea.edbc.org/."
color #cc00ff
type region
chr3 181403551 181455136 hg19_BBHM-150_chr3_181403551
chr3 181403691 181476186 hg19_BBCG_chr3_181403691
chr3 181403893 181455295 hg19_BBITL_chr3_181403893
chr3 181404243 181455400 hg19_BBAC_chr3_181404243
Or upload :
No file selected
Choose File
(2)
Please, upload a file fields terminated by "TAB". Example 1 (region) Example 2 (hist)
Submit
C View in genome browser.
Manage Custom Tracks
(3)
Existed Custom Tracks
View in SEA genome browser
+ Add Custom Tracks
Show 10 entries
Name
Description
Type
Position
Color
Items
test regions
super-enhancers of Huamn listed in SEA database http://sea.edbc.org/.
region
chr3:181403551-181449515
#cc00ff
8
Search:
First
Previous
1
Next
Last
D Visualization result with user custom file.
chr3(q26.33)
scale
chr3
CpGisland
SOX2-OT
SOX2-OT
SOX2-OT
LOC102724604
Super-Enhancer
TFBS
test regions
1 Mb
hg19
(result)
+ Add/Manage Custom Tracks (1)
(4)
Custom Tracks
test regions

Supplementary Figure S2. Custom visualization in SEA v. 3.0. (A) Selection of custom track for visualization. (B) Input of custom visual files. (C) View in genome browser. (D) Visualization result with user custom file.

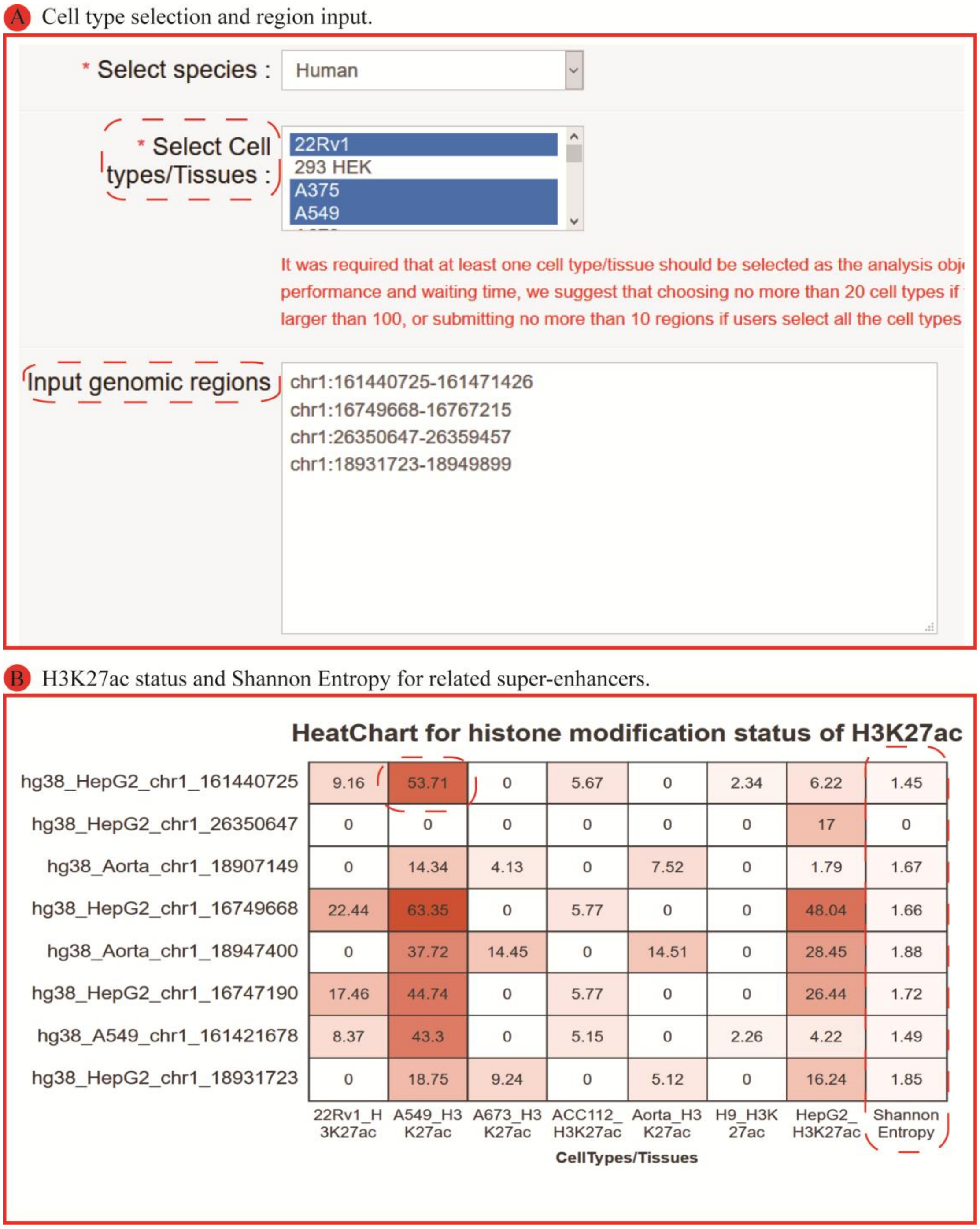

Supplementary Figure S3. Computation of Histone modification status and Shannon Entropy. (A) Cell type selection and region input. (B) H3K27ac status and Shannon Entropy for related super-enhancers.
